# CGX, a standardized herbal syrup, inhibits colon-liver metastasis by regulating the hepatic microenvironments in a splenic injection mouse model

**DOI:** 10.3389/fphar.2022.906752

**Published:** 2022-08-29

**Authors:** Sung-Bae Lee, Seung-Ju Hwang, Chang-Gue Son

**Affiliations:** Institute of Bioscience and Integrative Medicine, Daejeon University, Daejeon, Korea

**Keywords:** colon-liver metastasis, hepatic microenvironment, chuggan syrup (CGX), herbal medicine, hepatic steatosis

## Abstract

**Background:** Colon-liver metastasis is observed in approximately 50% of patients with colorectal cancer and is a critical risk factor for a low survival rate. Several clinical studies have reported that colon-liver metastasis is accelerated by pathological hepatic microenvironments such as hepatic steatosis or fibrosis. Chunggan syrup (CGX), a standardized 13-herbal mixture, has been prescribed to patients with chronic liver diseases, including fatty liver, inflammation and fibrotic change, based on preclinical and clinical evidence.

**Aim of the study:** In the present study, we investigated anti-liver metastatic the effects of CGX in a murine colon carcinoma (MC38)-splenic injection mouse model.

**Materials and methods:** C57BL/6N mice were administered with CGX (100, 200 or 400 mg/kg) for 14 days before or after MC38-splenic injection under normal and high-fat diet (HFD) fed conditions. Also, above experiment was repeated without MC38-splenic injection to explore underlying mechanism.

**Results:** The number of tumor nodules and liver weight with tumors were sup-pressed by preadministration of CGX in both normal and HFD fed mice. Regarding its mechanisms, we found that CGX administration significantly activated epithelial-cadherin (E-cadherin), but decreased vascular endothelial-cadherin (VE-cadherin) in hepatic tissues under MC38-free conditions. In addition, CGX administration significantly reduced hepatic steatosis, via modulation of lipolytic and lipogenic molecules, including activated adenosine monophosphate activated protein kinase (AMPK) and peroxisome proliferator activated receptor-alpha (PPARα).

**Conclusion:** The present data indicate that CGX exerts an anti-colon-liver metastatic property via modulation of hepatic lipid related microenvironments.

## Introduction

Metastasis is a critical process that determines the success or failure of cancer treatment; more than 60% of cancer-derived deaths are related to metastasis (Dillekås et al., 2019). Liver metastasis is most commonly observed by approximately 50% of colon cancer patients worldwide, while the average 5-years survival rate is notably decreased by 30%, even after surgical re-section of metastatic cancer (Simmonds et al., 2006; De Greef et al., 2016). The liver is a major target of metastasis in gastrointestinal-originated tumors because of the anatomical feature of blood flow called the portal vein (Tan, 2019). One clinical study showed that 35% of colon cancer patients who underwent a complete resection experienced liver-metastasis in 40 months (Sotelo et al., 2015).

In general, the risk of metastasis is associated with advanced levels of tumors, and an increasing number of studies have shown that the metastatic process starts at a very early stage of cancer, even when tumors are 5 mm in diameter (Kokubo et al., 2018; Takemoto et al., 2020). Researchers found that circulating tumor cells were observed in the blood of patients even after 30 days of resection of breast tumors (Sandri et al., 2010). These findings strongly suggest the importance of host-side determinants of cancer metastasis, called tumor microenvironments (TMEs) (Baghban et al., 2020). During colon-liver metastasis, colon cancer cells interact with various constituents of the liver microenvironments, such as extracellular matrix (ECM), cell adhesion molecules (CAMs), and many cellular components, including natural killer (NK) cells, Kupffer cell (KC), hepatic stellate cells (HSCs), and liver sinusoidal endothelial cells (LSECs) (Zeng et al., 2021).

Accordingly, liver microenvironments are undeniable components for metastasis. Several clinical studies have reported that pathological conditions such as hepatic steatosis, inflammation and fibrosis accelerate colon-liver metastasis (Kondo et al., 2016; Chen et al., 2021). These pathologic alterations in hepatic tissues make the liver microenvironment favorable for liver metastasis, as evidenced by animal studies (Matsusue et al., 2009; Li et al., 2020). We have previously reported that alcohol consumption aggravated the colon-liver metastatic seeding phase by upregulating the inter-cellular adhesion molecule 1 (ICAM1) and mild inflammatory changes in hepatic tissue (Im et al., 2016). These findings indicate that controlling liver microenvironments may be a strategy to reduce colon-liver metastasis.

Chunggan syrup (CGX), a multiherbal drug derived from traditional Korean medicine (TKM), has been prescribed for patients with chronic hepatic disorders. We previously observed anti-steatotic and anti-inflammatory effects *via* reducing hepatic lipid and oxidative stress in animal models (Shin et al., 2006; Kim et al., 2013; Park et al., 2013) as well as anti-fibrotic effects of CGX in a clinical trial (Joung et al., 2020). However, how the hepatoprotective effects of CGX affect the risk of colon-liver metastasis has not yet been studied.

In the present study, we aimed to investigate the pharmaceutical effects of CGX against colon-liver metastasis using a murine colon carcinoma (MC)38-splenic injection mouse model under both normal and fat diet conditions.

## Materials and methods

### Preparation and fingerprinting of CGX

**FIGURE 1:**
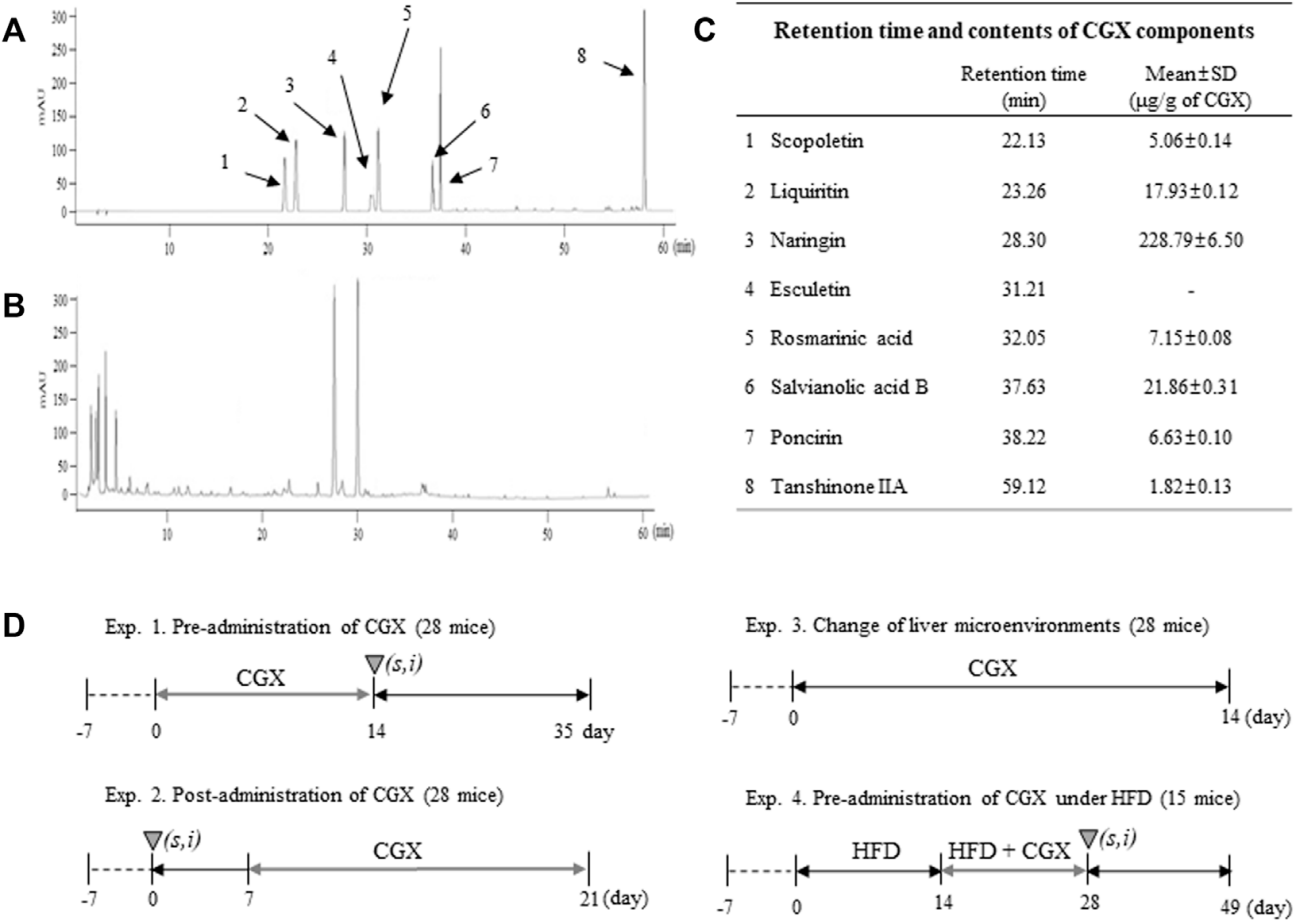
Fingerprinting of CGX and animal experimental designs. Eight reference compounds **(A)** in CGX **(B)** were analyzed using HPLC, and semiquantitative analysis was performed using their standard curves **(C)**. The mice (99 mice in total) were used according to four different experimental designs **(D)**.

CGX, which consisted of 13 kinds of herbal plants that complied with the Korean Pharmacopoeia standards, was manufactured by Kyung-bang Pharmacy according to the guidelines of the Korean Food and Drug Administration (Table 1). To confirm the chemical composition of CGX, ultrahigh-performance liquid chromatography tandem mass spectrometry (UHPLC-MS/MS, Thermo Scientific, Waltham, CA, United States) was performed according to a previous study (Kim et al., 2014). The chromatogram indicated that the main chemical components of CGX were scopoletin, liquiritin, naringin, esculetin, rosmarinic acid, salvianolic acid B, poncirin, and tanshinone IIA (Sigma Aldrich, MO, United States, Figures 1A–C).

**TABLE 1:** Components of CGX.

Herbal name	Scientific name	Amounts (g)
Artemisiae Capillaris Herba	*Artemisia capillaris* Thunberg	5
Amydae Carapax	*Amyda sinensis* (Wiegmann)	5
Raphanus Seed	*Raphanus sativus* L	5
Atractylodis Rhizoma Alba	*Atractylodes macrocephala* Koidz	3
Hoelen	*Poria cocos* Wolf	3
Alismatis Rhizoma	*Alisma orientalis (Sam.)* Juzepczuk	3
Atractylodis Rhizoma	*Atractylodes chinensis* Koidzumi	3
Salviae Miltiorrhizae Radix	*Salvia miltiorrhiza* Bunge	3
Polyporus	*Polyporus umbellatus* Fries	2
Aurantii Immaturus Fructus	*Poncirus trifoliate* Rafin	2
Amomi Fructus	*Amomum villosum* Lour	2
Glycyrrhizae Radix	*Glycyrrhiza uralensis* Fisch	1
Aucklandiae Radix	*Aucklandia lappa* Decne	1

### Cell culture

Murine colon carcinoma (MC)38 was purchased from Kerafast (MA, United States) and cultured at 37°C in a 5% CO_2_ chamber in Dulbecco’s modified Eagle medium (DMEM) with 10% fetal bovine serum (FBS) and 1% penicillin/streptomycin solution (WelGENE Inc., Kyeong-book, Korea).

### Animals and experimental designs

Male C57BL/6N mice (8 weeks of age, 22–25 g) purchased from Daehan Biolink (Choong-book, Korea) were housed in a room maintained at 22 ± 2°C under a 12 h light/12 h dark cycle with a free-accessing standard diet (Zeigler Co. PA, United States) and tap water. The animal experiments were conducted in accordance with the Guide for the Care and Use of Laboratory Animals prepared by the US National Institutes of Health and were approved by the Institutional Animal Care and Use Committee of Daejeon University (DJUARB 2022-002). A total of 99 mice were used according to four different experimental de-signs (Figure 1D).

In this study, colon-liver metastasis was performed *via* splenic injection of MC38 cells according to a previous study (VanSaun et al., 2009). Briefly, the spleen was exteriorized by an incision of the left upper abdomen under anesthetization using a ketamine/xylazine mixture (90/10 mg/kg for one mouse). Then, MC38 cells (1 × 10^5^) in 100 μL of Dulbecco’s phosphate-buffered saline (DPBS, WelGENE Inc., Kyeong-book, Korea) were slowly injected into the spleen. To allow the cells to arrive at the liver, the needle was left without removal for 90 sec, followed by removal of the spleen and ligation of the splenic vein/artery.

### Animal experiment 1

In total, 28 mice were randomly divided into four groups (each with seven mice), and all mice were orally administered water or CGX (100, 200 or 400 mg/kg) for 14 days. For all mice, a splenic injection of MC38 cells (1 × 10^5^/mouse) was performed evenly one by one for four groups on the day of the final administration of CGX. After 21 days without any manipulation, the mice were sacrificed in a CO_2_ chamber. The liver weights and numbers of nodules were evaluated by two researchers who were blinded to group allocations.

### Animal experiment 2

In total, 28 mice were administered a splenic injection of MC38 cells (1 × 10^5^/mouse) and allowed to recover for 7 days. Then, the mice were randomly divided into four groups (each with seven mice), and all mice were orally administered water or CGX (100, 200 or 400 mg/kg) for 14 days. Twenty-one days after the MC38 injection, all mice were sacrificed in a CO_2_ chamber, and the liver weights and numbers of nodules were evaluated by two re-searchers who were blinded to group allocations.

### Animal experiment 3

In total, 28 mice were randomly divided into four groups (each with seven mice), and all mice were orally administered water or CGX (100, 200 or 400 mg/kg) for 14 days. Body weights were measured once a week. After 14 days without splenic injection of MC38 cells, mice were sacrificed in a CO_2_ chamber. The liver and three abdominal fats (epididymal, retroperitoneal and visceral fat) were respectively weighed, and then the blood and liver were acquired for PCR, ELISA, western blotting, and histological analysis.

### Animal experiment 4

In total, 15 mice were randomly divided into three groups (each with five mice): control (normal chow diet/water), HFD (high fat diet/water) and HFD + CGX (high fat diet/CGX 400 mg/kg). The HFD and HFD + CGX groups were free-fed a 60% high-fat diet (HFD, Research Diets Inc. NJ, United States) for 4 weeks. Compositions of normal chow diet and HFD were described in Table 2. Beginning 2 weeks after feeding HFD, mice were orally administered water or CGX 400 mg/kg for 14 days. For all mice, splenic injection of MC38 cells (1 × 10^5^/mouse) was performed evenly one by one for four groups on the day of final administration of CGX. After 21 days without any manipulation, the mice were sacrificed in a CO_2_ chamber. The liver weights and numbers of nodules were evaluated by two researchers who were blinded to group al-locations.

**TABLE 2:** Composition of normal chow diet and 60% high-fat diet.

Composition	Normal diet		High-fat diet	
	g %	kcal %	g %	kcal %
Carbohydrate	72	73	26	20
Protein	18	18	26	20
Fat	4	9	34	60
kcal/g	4.0		5.2	

### Histological analysis in hepatic tissue

To evaluate histology and fat accumulation, hepatic tissues were fixed in 10% formalin solution. The formalin in tissue was removed by storing in 10, 20, and 30% sucrose solution in a stepwise manner, and then cryosections of tissue were performed using a Leica CM3050 cryostat (Leica Microsystems). The sections were stained with hematoxylin and eosin (H&E, Sigma Aldrich, MO, United States) and Oil red O (Sigma Aldrich, MO, United States). The staining slides were visualized using microscope (Leica, Wetzlar, Germany) at 100× (H&E) and ×200 (Oil red O) magnifications, respectively.

### Fat composition and lipid levels in the liver tissue

The fat composition was measured using dual-energy X-ray absorptiometry (DEXA) with an InAlyzer (Medikors Co., Seongnam, Korea). Briefly, large leaves of liver tissue were placed on the scanner bed and then scanned according to the instructions for operating the InAlyzer system. The fat mass in liver tissue was calculated as 100% of the control group, and the percentage was compared other groups. TG and TC levels were determined in normal liver tissue for normal diet feeding mice or liver tissue containing tumor for HFD feeding mice using commercial kits (ASAN pharmacy, Seoul, Korea) according to a previous method (Carr et al., 1993).

### Western blotting

50 mg of hepatic tissues were dissolved in Radioimmunoprecipitation assay (RIPA) buffer and adjusted to a concentration of 1 mg/ml using the Bio-Rad protein assay reagent (Hercules, CA, United States), and then 25 μL of samples were separated by seven or 15% poly-acrylamide gel electrophoresis and transferred to polyvinylidene fluoride (PVDF) mem-branes. After blocking in 5% skim milk or bovine serum albumin (BSA) for 1 h, the mem-branes were probed with primary antibodies against pAMPK (Cell Signaling, Cambridge, United Kingdom), PPARα (Abcam, Cambridge, United Kingdom), E-cadherin (Cell Signaling, Cambridge, United Kingdom), VE-cadherin (Abcam, Cambridge, United Kingdom) and actin (Thermo Fisher Scientific, PA, United States) at 4°C overnight. Then, the membranes were washed three times for 15 min each and incubated for 2 h with HRP-conjugated anti-rabbit or anti-mouse secondary antibody. Blots were developed using an enhanced chemiluminescence (ECL) advanced kit and visualized using UV fluorescence autoexposure in the Fusion Solo S system (VILBER ROURMAT, France). Protein expression was semiquantified using ImageJ software (NIH). In the present study, Western blot data were presented with one of them as representative image.

### Real-time PCR

Total RNA from the hepatic tissues was extracted using QIAzol reagent (Qiagen, Hil-den, Germany). Complementary DNA (cDNA) was synthesized using a High-Capacity cDNA Reverse Transcription Kit (Ambion, TX, United States). Real-time PCR was 40 cycles performed on diacylglycerol acyltransferase 1, 2 (DGAT one and 2), hydroxy-methyl-glutaryl coenzyme A reductase (HMGCR), hormone-sensitive lipase (LIPE), monoglyceride lipase (MGLL), acetyl-coenzyme A acyltransferase 2 (ACAA2) and β-actin under the condition below; denaturation (95°C, 15 sec), annellation (60°C, 15 sec) and extension (72°C, 1 min) using SYBR Green PCR Master Mix (Applied Biosystems, CA, United States) and Rotor-Gene Q (Qiagen, Hilden, Germany). All CT-values for above genes were normalized by β-actin and presented as fold-value. The primer sequences used in the gene expression analyses were summarized in Table 3.

**TABLE 3:** Primer sequences.

Primer name	Forward	Reverse
DGAT1	CAT​CAT​GTT​CCT​CAA​GCT​TTA​TTC​CT	ACT​GAC​CTT​CTT​CCC​TGT​AGA​GAC​A
DGAT2	CAC​CCT​GAA​GAA​CCG​CAA​A	RCTGCTTGTATACCTCATTCTCTCCAA
HMGCR	GGG​CCC​CAC​ATT​CAC​TCT​T	GCC​GAA​GCA​GCA​CAT​GAT​CT
LIPE	ATG​AAG​GAC​TCA​CCG​CTG​ACT​T	CGG​ATG​GCA​GGT​GTG​AAC​T
MGLL	TGG​TGT​CGG​ACT​TCC​AAG​TTT	GAG​TGG​CCC​AGG​AGG​AAG​AT
ACAA2	CTC​TGC​CAC​CGA​TTT​AAC​TGA​A	CAT​GAC​ATT​GCC​CAC​GAT​GA

DGAT1 and 2, diacylglycerol acyltransferase 1 and 2; HMGCR, hydroxy-methyl-glutaryl coenzyme A reductase; LIPE, hormone-stimulated lipase; MGLL, monoglyceride lipase; ACAA2, acetyl-coenzyme A acyltransferase 2.

### Statistical analysis

The results are expressed as the means ± standard deviation (SD, *n* = 3∼7). All statistical significance among the groups was analyzed by one-way ANOVA followed by post hoc multiple comparisons (Tukey HSD) using Statistical Package for the Social Sciences (PRISM). For all analyses, *p < 0.05 or **p < 0.01 indicated statistical significance of each group compared to another group.

## Results

### Effects of pre- and post-administration of CGX on colon-liver metastasis

**FIGURE 2:**
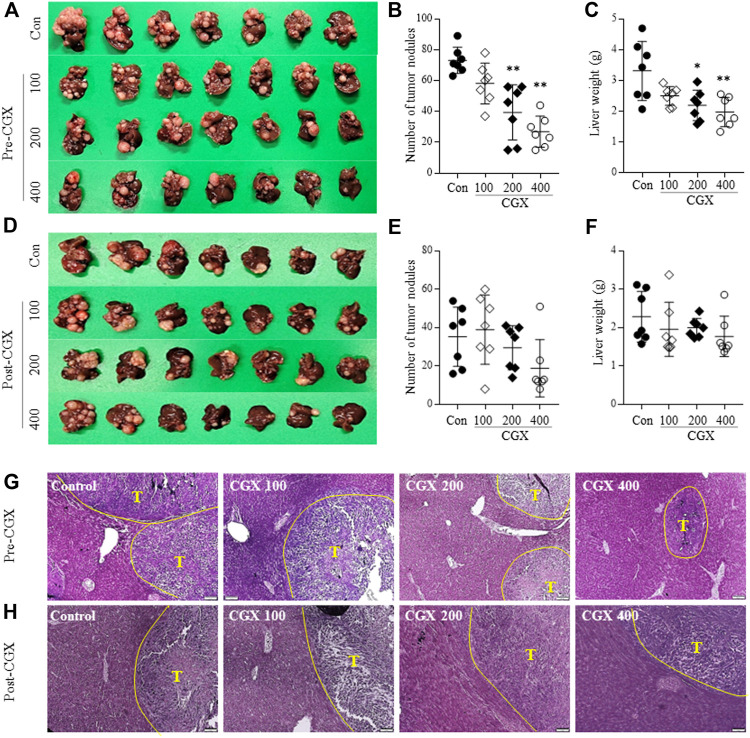
Anti-metastatic effects of pre- and postadministration of CGX. Mice were given pre- **(A–C)** and **(G)** or postadministered **(D–F)** CGX (100, 200 or 400 mg/kg, 14 days) *via* MC38-splenic injection. After 21 days of MC38-splenic injection (1 × 10^5^/mouse), the numbers of tumor nodules and liver weight were measured. H&E staining was performed in liver tissue and visualized at ×100 magnifications **(G–H)**. The data are expressed as the means ± SD (*n* = 7). *p < 0.05 or **p < 0.01 indicates statistical significance compared to the control group. T; Tumor nodule.

Using an MC38-splenic injection mouse model, we evaluated the anti-liver metastatic effects of both pre- or postadministration of CGX for 14 days. The preadministration of CGX significantly reduced the numbers of metastatic tumor nodules in the liver compared to the control group (*p* < 0.01, Figures 2A,B), along with a significant decrease in liver weights (*p* < 0.05 or 0.01, Figure 2C). The postadministration of CGX also showed a slight re-duction in these parameters, but the difference did not reach statistical significance (*p* > 0.05, Figures 2D–F). The anti-liver metastatic effects of the preadministration of CGX were consistent with hematoxylin and eosin (H&E) staining in hepatic tissue (Figures 2G,H).

### Effects of CGX on lipid content-related microenvironments in hepatic tissue

**FIGURE 3:**
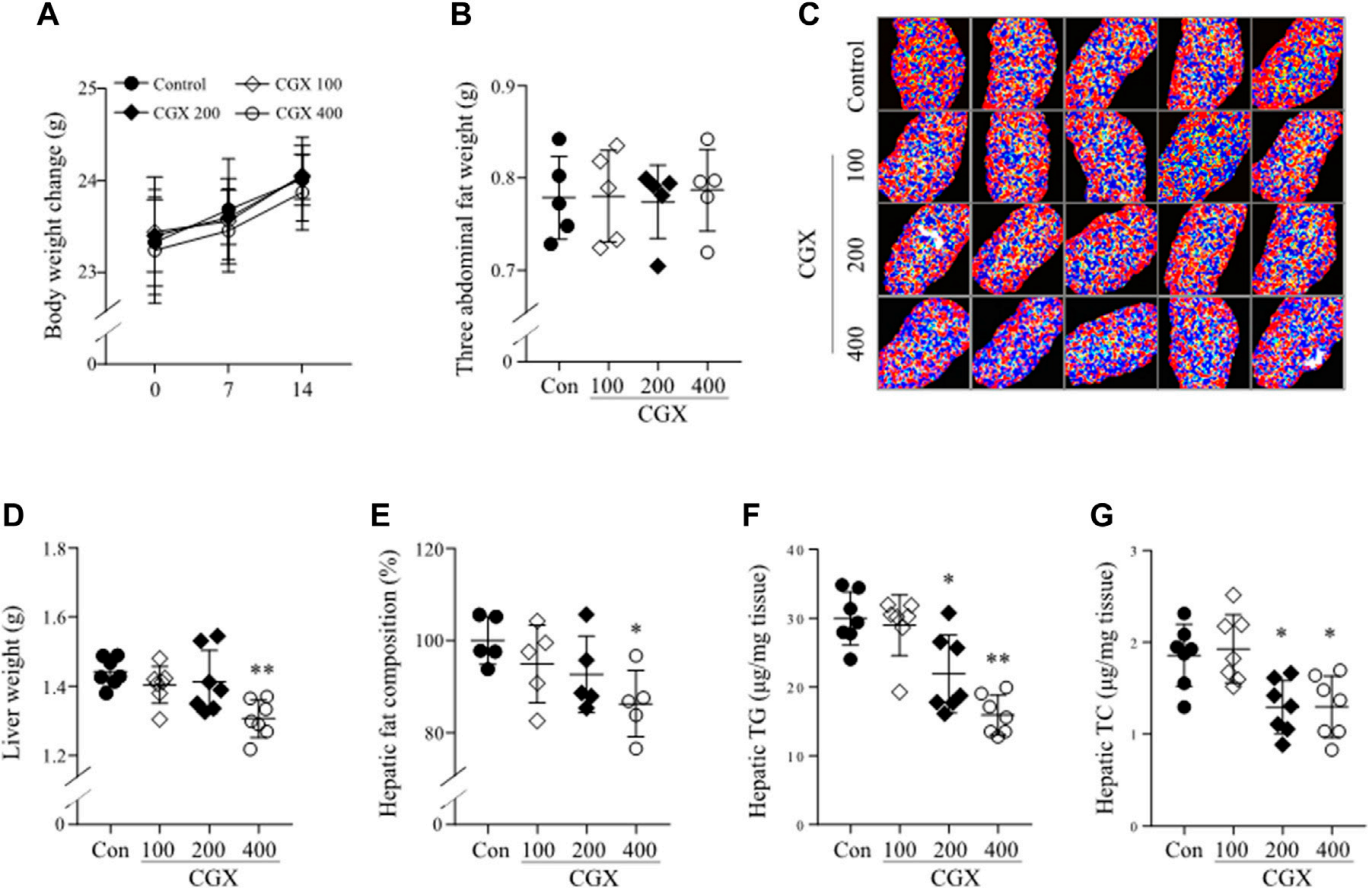
Effects of CGX on lipid components in body and hepatic tissue. Under MC38-free conditions, mice were administered with CGX (100, 200 or 400 mg/kg, 14 days). Body weight **(A)** was measured once a week, and the three abdominal fats (epididymal, retroperitoneal and visceral fat, **(B)** and liver **(D)** were weighed after sacrifice. The hepatic fat composition was measured using a DEXA analyzer and expressed as a relative value compared to the control group **(C,E)**. Hepatic TG and TC were determined using commercial kits **(F,G)**. The data are expressed as the means ± SD (*n* = 5 or 7). *p < 0.05 or **p < 0.01 indicates statistical significance compared to the control group. DEXA; dual energy X-ray absorptiometry, TC; total cholesterol, TG; triglyceride.

To investigate how CGX affected the anti-liver metastatic effects, we examined the changes in whole body and hepatic microenvironments using MC38-free conditions. There was no change in body and abdominal fat weights by CGX (*p* > 0.05, Figures 3A,B), while CGX administration (especially 400 mg/kg) significantly reduced liver weights compared to the control group (*p* < 0.01 Figure 3D). This result was confirmed by fat composition imaging (*p* < 0.05, Figures 3C,E), quantity of triglyceride (TG, *p* < 0.05 or 0.01, Figure 3F) and total cholesterol (TC, *p* < 0.05, Figure 3G) in hepatic tissue.

### Effects of CGX on adherent and lipid-related molecules in hepatic tissue

**FIGURE 4:**
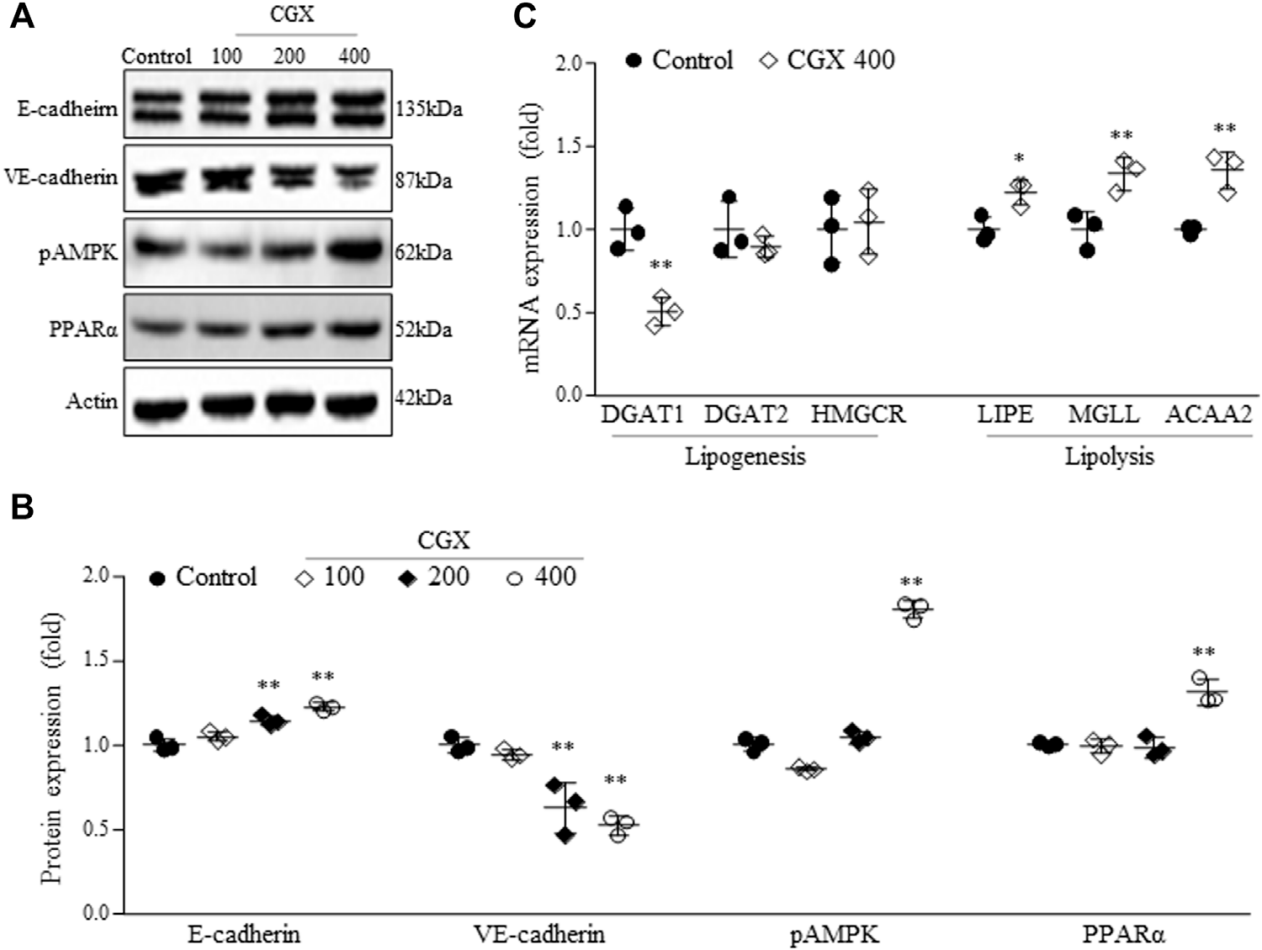
Effects of CGX on cell adhesion- and lipid metabolism-related molecules. The protein **(A,B)** and mRNA levels **(C)** were determined using western blotting and PCR, and normalized to β-actin, respectively. The data are expressed as the means ± SD (*n* = 3). *p < 0.05 or **p < 0.01 indicates statistical significance compared to the control group. E-cadherin; epithelial cadherin, VE-cadherin; vascular endothelial cadherin, pAMPK; phospho adenosine monophosphate kinase, PPARα; peroxisome proliferator-activated receptor alpha, DGAT1 and two; diacylglycerol acyltransferase 1 and 2, HMGCR; hydroxy-methyl-glutaryl coenzyme A reductase, LIPE; hormone-sensitive lipase, MGLL; monoglyceride lipase, ACAA2; acetyl-coenzyme A acyltransferase 2.

To investigate the mechanisms underlying the anti-liver metastatic effects of CGX, we examined the two-representative cell-adherent molecules. CGX administration activated epithelial-cadherin (E-cadherin) in a dose-dependent manner but suppressed vascular endothelial-cadherin (VE-cadherin) at the protein level in hepatic tissues (*p* < 0.01, Figures 4A,B). In addition, we confirmed that CGX administration (especially 400 mg/kg) modulated two typical lipid metabolism-related molecules, phospho adenosine monophosphate kinase (pAMPK) and peroxisome proliferator-activated receptor alpha (PPARα) in hepatic tissue under general condition (*p* < 0.01, Figures 4A,B). These results were supported by analyses of mRNA expression for both lipogenesis genes (*p* < 0.01 in DGAT1) and lipolysis genes (*p* < 0.05 or *p* < 0.01 in LIPE, MGLL and ACAA2, Figure 4C).

### Effects of preadministration with CGX on colon-liver metastasis under high-fat diet conditions

**FIGURE 5:**
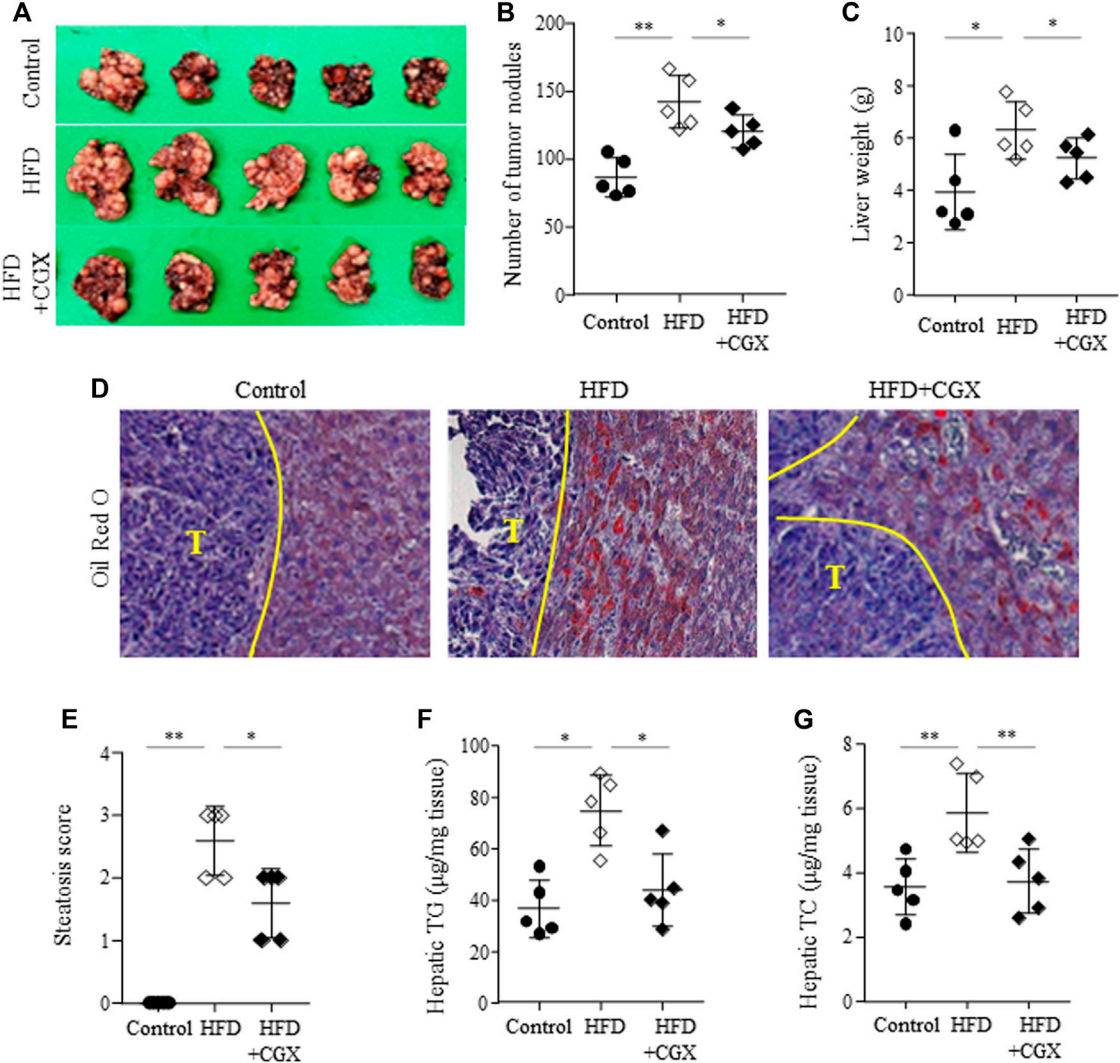
Effects of anti-metastatic effects of CGX in the HFD-induced metastasis model. Beginning 2 weeks after HFD feeding, the mice were orally administered water or CGX (400 mg/kg) for 14 days. A splenic injection of MC38 cells (1 × 10^5^/mouse) was performed on the day of the final administration of CGX. The liver weights and numbers of nodules were evaluated by two researchers who were blinded to group allocations **(A–C)**. Oil red O staining was performed in the liver tissue and visualized at ×200 magnifications **(D)**. Steatosis area was scored according to the nonalcoholic steatohepatitis (NASH) clinical research network scoring system **(E)**. Hepatic TG and TC were determined using common kits **(F)** and **(G)**. The data are expressed as the means ± SD (*n* = 5). *p < 0.05 or **p < 0.01 indicates statistical significance compared to each group. T; Tumor nodule.

We confirmed the anti-liver metastatic effects of CGX (400 mg/kg) under high-fat diet (HFD)-induced fatty liver conditions. HFD dramatically accelerated the liver metastasis of colon tumors, as measured by both the number of tumor nodules and liver weight (*p* < 0.01). Then, these were effects significantly attenuated by the administration of CGX (*p* < 0.05, Figures 5A–C). As expected, the TG and TC contents in hepatic tissues were significantly reduced in CGX (*p* < 0.05, Figures 5F,G), which was confirmed by Oil Red O staining (*p* < 0.05, Figures 5D,E).

## Discussion

Although therapeutic strategies for cancer have advanced, metastasis remains the main cause of cancer-related death. Metastasis is a multistep process of intravasation, survival in blood, extravasation and colonization of cancer cells (Sahai, 2007). The interaction be-tween the tumor and the TME is known to play a key role in the success or failure of those metastatic processes (Fontebasso and Dubinett, 2015; Chandra et al., 2021). Accordingly, many recent studies emphasize the importance of the TME as a therapeutic target (Steeg, 2016; Fares et al., 2020). In addition to TME in tumor-originated sites, the circumstance of liver tissue (i.e., the metastatic target-legion of colorectal tumors) also markedly affects the end step of metastasis likely the acceleration of liver metastasis in inflammation, steatosis, or fibrotic change (Rossetto et al., 2019). Furthermore, symptomatic dormant cancer cells in the liver disseminated from the primary lesion could be reactivated to proliferate into histologic metastasis under certain alterations of hepatic microenvironments (Llovet et al., 2021). From this aspect, we evaluated the anti-colon-liver metastatic effects of CGX which has been proven to exert hepatoprotective effects in many studies. Preadministration of CGX resulted in a significant reduction in liver metastasis among cancer cells (Figures 2A–C). Even though the postadministration of CGX also showed a similar pattern, the effect was weaker than the preadministration results of CGX (Figures 2D–F). These findings indicate that CGX exerts preventive but not therapeutic effects against colon-liver metastasis.

In the present study, we adapted a splenic injection-derived colon-liver metastasis model established previously (VanSaun et al., 2009). The experimental model mimics the clinical phenotype of liver metastasis, in which most tumor cells in the gastrointestinal tract mainly enter the portal vein and spread into liver tissue (Kok et al., 2021). Colon and liver metastasis occur *via* the pas-sage portal vein, which cancer cells can reach and contact LSECs (Vidal-Vanaclocha et al., 1993). CAMs in LSECs play key roles in calling the tumor cells inside the sinusoid bloodstream into hepatic tis-sue for extravasation (Paschos et al., 2009; Leong et al., 2014). CAMs in endothelial cells are also implicated in both the detachment and settlement of cancer cells in distant sites (Harjunpää et al., 2019). We found that CGX treatment significantly down- and upregulated two typical metastasis-related CAMs, VE-cadherin and E-cadherin, in liver tissue (Figures 4A,B). In fact, clinical studies have reported a significant correlation between the risk of liver metastasis and high blood levels of VE-cadherin but low levels of E-cadherin (Velikova et al., 1998; Dymicka-Piekarska and Kemona, 2009; Okugawa et al., 2012; Rochefort et al., 2017). E-cadherin works especially at cell-cell junctions, so it protects against the extravasation of tumor cells into tissues of secondary sites (Onder et al., 2008; Samanta and Almo, 2015). Another group showed that cancer cells need to incorporate with VE-cadherin into vascular endothelial cells in the initial step of extravasation using a VE-cadherin knockout model, (Hamilla et al., 2014). Based on these facts, our data indicate that CGX may inhibit the extravasation step of colon cancer in hepatic sinusoidal vessels. Additionally, we confirmed that CGX has no effects on metastatic parameters such as intercellular adhesion molecule 1 (ICAM1), vascular cell adhesion protein 1 (VCAM1) and E-selectin (data not shown).

Components of liver including hepatocytes, KCs and HSCs play key roles colon-liver metastasis. Especially, fat excessive accumulation in hepatocyte lead to hepatic inflammation which allow those tumor cells to escape immune surveillance to colonize liver tissue (Brodt, 2016). Furthermore, it leads to activate HSCs which promote tumor growth and metastasis in liver (Matsusue et al., 2009). In clinic, nonalcoholic fatty liver disease (NAFLD) is known as an accelerator of liver metastasis, increasing the metastatic incidence by 2.5-fold in patients with colorectal cancer (Lv and Zhang, 2020). Our 28 days HFD feeding model showed dramatically increase in body weight (Supplementary Figure S1C) and mild hepatic steatosis (Figures 5D–G) which are accordance with our previous study (Hwi-Jin Im et al., 2020). Also, our HFD model severely accelerated colon-liver metastasis; the number of tumor nodules and liver weight was approximately 1.6-fold higher in the NAFLD model than in the normal diet model (Figure 5A). Then, we confirmed the anti-metastatic effects of CGX in both normal and HFD-fed mouse models. Interestingly, anti-metastatic effects showed in preadministration of CGX but not postadministration (Figures 2, 5). These effects are explained by changing the liver environment into a state that inhibits metastasis before the tumor invades the liver. Also, our results are supported by role and importance of host microenvironment in metastasis based on seed and soil theory (Brodt, 2016; Liu et al., 2017). In fact, our previous animal and clinical studies reported the hepatoprotective effects of CGX against hepatic inflammation and fibrosis as well as HFD or alcohol-induced hepatic steatosis (Shin et al., 2006; Kim et al., 2013; Park et al., 2013; Joung et al., 2020). These results indicate that CGX prevent metastasis *via* modulation of liver environments, especially hepatic lipid.

Regarding the anti-liver metastatic effects of CGX in the present study, we anticipated that the modulation of hepatic lipid profiles might contribute to the reduction of colon-liver metastasis (Figures 3, 4). Tumor cells require an aberrantly high supply of energy from carbohydrates and lipids for fast growth; therefore, alteration of lipid contents and metabolism is considered one of the hallmarks of tumors (Beloribi-Djefaflia et al., 2016; Jeong et al., 2021). Recent studies have showed that hepatic lipids promote liver metastasis *via* excessive increased metabolism in tumor cells (Li et al., 2020). Fatty liver conditions are also known to alter CAMs, including E-selectin, ICAM1 and VCAM1, into favorable metastasis directions in hepatic tissues (Lin et al., 2010; Oktay Bilgir, 2014). In particular, several research groups showed that VE-cadherin in liver sinusoid endothelial cells was activated in patients with NAFLD (Kan et al., 2016), and steatotic hepatocytes suppress E-cadherin compared to normal hepatocytes (Pan et al., 2015). These facts support that CGX’s inhibition of liver metastasis involves actions on hepatic lipid contents, thereby leading to modulations of VE-cadherin and E-cadherin in hepatic tissue.

CGX is an herbal syrup composed of multiple medicinal plants and is standardized as eight chemical compounds, including scopoletin, liquritin, naringin, esculetin, rosmarinic acid, salvianolic acid B, poncirin and tanshinone IIA (Figures 1A–C). Some data using the above compounds support our results. In addition, naringin and esculetin inhibited lipid accumulation in hepatocytes *via* the modulation of gut microbiota in mouse models (Park et al., 2017; Mu et al., 2020). In the present study, although active compounds for anti-metastatic effects may be predicted *via* previous studies, it further need to identifying active compounds of CGX which have antimetastatic effects.

Taken together, our findings indicate that CGX has an anti-colon-liver metastatic effect. Its underlying mechanisms involve the modulation of liver microenvironments, including hepatic lipids and their mediated adhesion molecules (VE-cadherin and E-cadherin), and these effects is preventive but not therapeutic.

## Data Availability

The original contributions presented in the study are included in the article/Supplementary Materials, further inquiries can be directed to the corresponding author.

## Supplementary material

Supplementary Figure 1 MC38 and AML12 cells seeded in 96-well plate (2×10^3^/well), 60-mm culture dish (1×10^5^/dish) or 100-mm culture dish (3×10^5^/dish) for determination of cell viability, mRNA or protein expression. The cells were exposed CGX (1, 10, 100 or 1000 μg/mL) for 24 h. Cell viabilities were measured using cell-based assay kit (EZ-Cytox, DoGenBio, Seoul, Korea, **A**). In vitro model, PCR on Ki67 **(B)** and western blot analysis on E-cadherin **(C, D)** were performed according to method of main article. In vivo, western blot analysis on E-cadherin and VE-cadherin in individual-hepatic tissue was performed according to method of main article **(E)**. Mice were weighed on the 28 days of HFD feeding **(F)**. **p* < 0.05 or ***p* < 0.01 indicates statistical significance compared to the control group. ^##^ *p* < 0.01 indicates statistical significance compared to the HFD group. AML1; alpha mouse liver 12, CGX; chunggan syrup, E-cadherin; epithelial cadherin, HFD; high-fat diet, VE-cadherin; vascular endothelial cadherin.
