# Supplementary figures and images for: CGX, a standardized herbal syrup, inhibits colon-liver metastasis by regulating the hepatic microenvironments in a splenic injection mouse model
Source: Front Pharmacol. 2022 Aug 29;13:906752. doi: 10.3389/fphar.2022.906752 (PMC9465806; doi:10.3389/fphar.2022.906752)

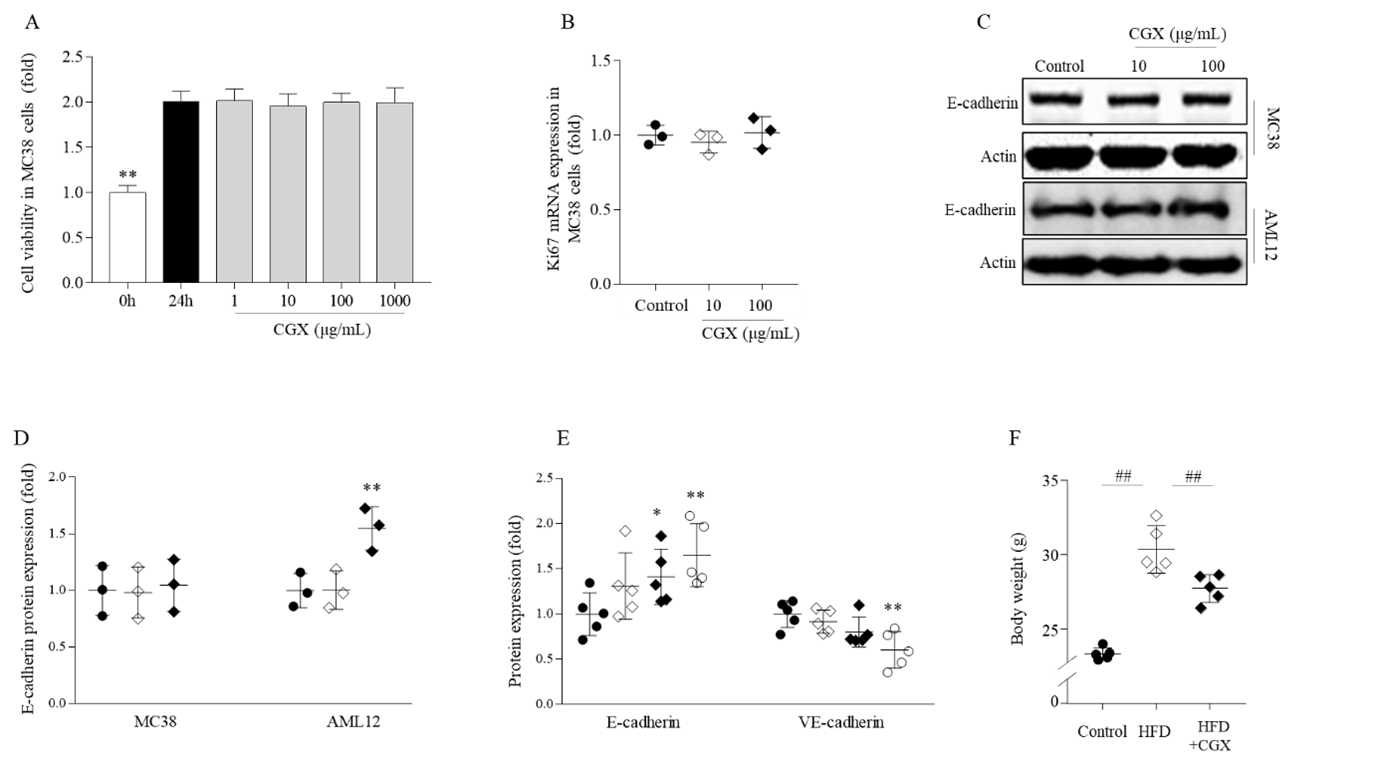

Supplement: Supplementary file 1 [file Image1.tif]
