# Supplementary material for: CGX, a standardized herbal syrup, inhibits colon-liver metastasis by regulating the hepatic microenvironments in a splenic injection mouse model
Source: Front Pharmacol. 2022 Aug 29;13:906752. doi: 10.3389/fphar.2022.906752 (PMC9465806; doi:10.3389/fphar.2022.906752)
Supplement: Supplementary file 2 [file Image1.DOCX]

**
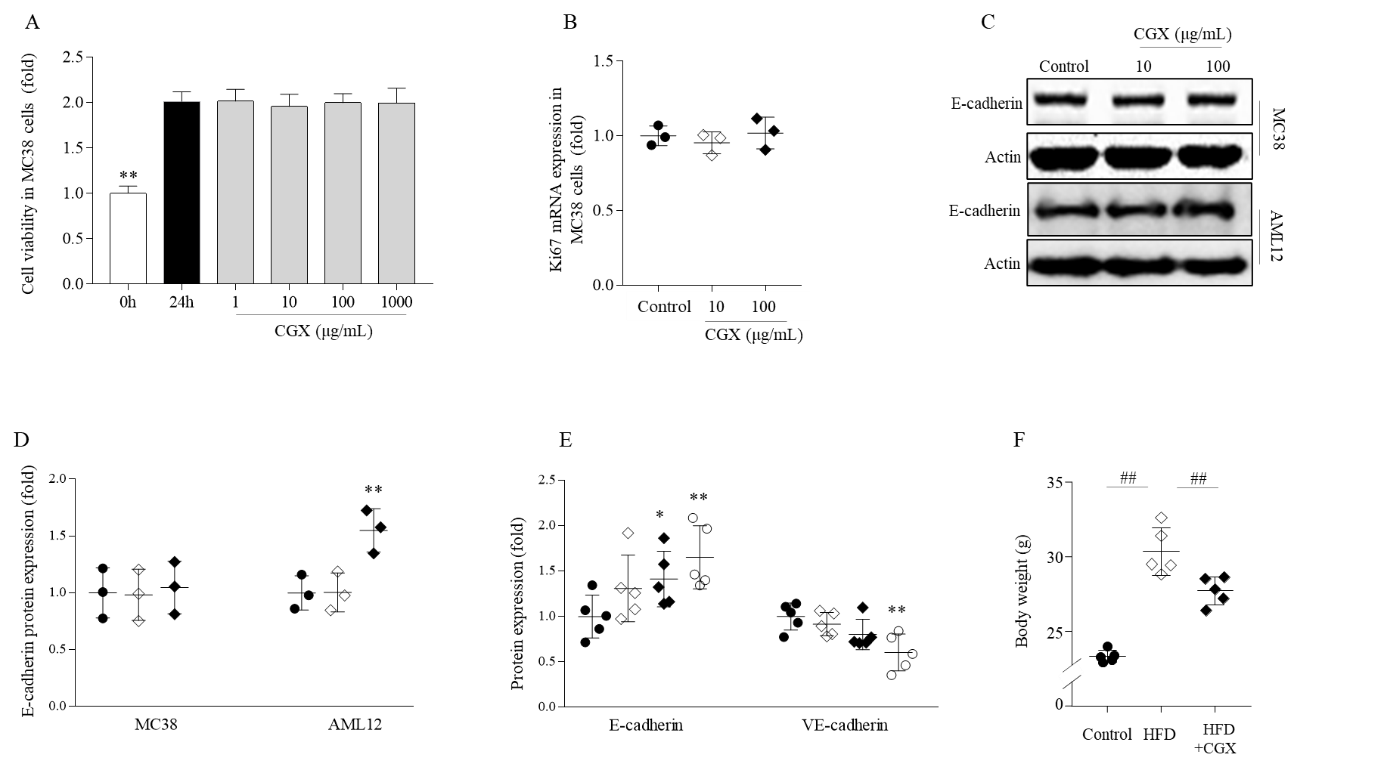
Supplementary figure 1.** MC38 and AML12 cells seeded in 96-well plate (2×10^3^/well), 60-mm culture dish (1×10^5^/dish) or 100-mm culture dish (3×10^5^/dish) for determination of cell viability, mRNA or protein expression. The cells were exposed CGX (1, 10, 100 or 1000 μg/mL) for 24 h. Cell viabilities were measured using cell-based assay kit (EZ-Cytox, DoGenBio, Seoul, Korea, A). In vitro model, PCR on Ki67 (B) and western blot analysis on E-cadherin (C, D) were performed according to method of main article. In vivo, western blot analysis on E-cadherin and VE-cadherin in individual-hepatic tissue was performed according to method of main article (E). Mice were weighed on the 28 days of HFD feeding (F). **p* < 0.05 or ***p* < 0.01 indicates statistical significance compared to the control group. ##*p* < 0.01 indicates statistical significance compared to the HFD group. AML1; alpha mouse liver 12, CGX; chunggan syrup, E-cadherin; epithelial cadherin, HFD; high-fat diet, VE-cadherin; vascular endothelial cadherin.
